# The prevalence of perioperative iron deficiency anaemia in women undergoing caesarean section—a retrospective cohort study

**DOI:** 10.1186/s13741-022-00268-x

**Published:** 2022-08-04

**Authors:** Alicia T. Dennis, Marissa Ferguson, Sarah Jackson

**Affiliations:** 1 The Royal Women’s Hospital, Locked Bag 300, Corner Grattan St. & Flemington Rd., Parkville, Victoria 3052 Australia; 2 School of Medicine, Faculty of Health, Deakin University, Geelong, Australia; 3 Departments of Critical Care, Obstetrics & Gynaecology, and Pharmacology Faculty of Medicine, Dentistry and Health Sciences, University of Melbourne, Melbourne, Australia; 4 Austin and Repatriation Medical Centre, 145 Studley Rd., Heidelberg, Victoria 3084 Australia; 5 Department of Critical Care, University of Melbourne, Melbourne, Australia; 6 Monash University, Melbourne, Australia; 7 Barwon Health, Ryrie Street, Geelong, Victoria 3220 Australia

**Keywords:** Pregnancy, Caesarean section, Iron deficiency, Anaemia, Perioperative medicine, Maternal morbidity and mortality, Neonatal morbidity and mortality, Haemoglobin

## Abstract

**Background:**

Caesarean section is a common surgery, with almost 23 million procedures performed globally each year. Postpartum haemorrhage, in association with caesarean section surgery, is a leading global cause of maternal morbidity and mortality. Perioperative iron deficiency anaemia is a risk factor for intraoperative bleeding. Therefore, anaemia is an important and modifiable risk factor for bleeding during caesarean section surgery. Recent recommendations advise that all preoperative patients with anaemia (defined as haemoglobin concentration (Hb) < 130 g/L), regardless of sex, be assessed and treated to normalise haemoglobin levels. It is unclear how this recommendation translates to pregnant women where the World Health Organization (WHO) defines anaemia at a much lower threshold (Hb < 110 g/L). We aimed to determine the prevalence, and characterization, of Hb levels < 130 g/L perioperatively in women undergoing caesarean section.

**Method:**

We conducted a retrospective cohort study of 489 consecutive women who underwent caesarean section over a 12-week period, in a single-centre tertiary referral maternity unit in Australia. We calculated the proportion of women who were anaemic (Hb < 130 g/L) at four time points—first hospital appointment, third trimester, preoperatively and on discharge from hospital. The proportion of women who were iron deficient (ferritin level < 30 μg/L) at their first hospital appointment was determined.

**Results:**

Haemoglobin was measured in 479 women. Ferritin was measured in 437 of these women. The mean (SD) Hb at the first hospital appointment, third trimester, preoperatively, and postoperatively on discharge was 126.7 (11.4) g/L, 114.6 (10.6) g/L, 124.1 (12.4) g/L, and 108.0 (13.6) g/L respectively. Iron deficiency was present in 148 (33.9%) women at their first hospital appointment; 107 of 248 (43.1%) women with anaemia and 41 of 189 (21.7%) with no anaemia. 29 women were found to have moderate anaemia (Hb 80−109 g/L) with 18 of these 29 (62.1%) women having iron deficiency. Only 68 (45.9%) women with iron deficiency at their first hospital appointment received treatment. The prevalence of anaemia classified as Hb < 130 g/L versus the WHO classification of Hb < 110 g/L from all causes was 57.4% versus 6.1% at first hospital appointment, 94% versus 26.1% in third trimester, and 66.0% versus 12.2% preoperatively. Postoperatively at least 40% of women had Hb < 130 g/L on hospital discharge versus at least 23% of women using WHO definition of Hb < 110 g/L. Of the 112 women with hospital discharge Hb < 110 g/L, 35 (31.3%) women were iron deficient at their first hospital appointment.

**Conclusion:**

Over one in three women were iron deficient at their first hospital appointment. 62% of women with moderate anaemia (Hb 80–109 g/L) also had iron deficiency. At least four in 10 women were anaemic (Hb < 130 g/L) on hospital discharge. Less than half of the women with anaemia were treated. Our data suggests that 30% of postoperative anaemia may be prevented with intensive treatment of iron deficiency in early pregnancy. Large prospective studies, are needed to determine outcomes after caesarean section in women, stratified by preoperative Hb and ferritin levels. The prevalence of anaemia in our data suggests it is a moderate public health problem.

## Background

Caesarean section is one of the most common operations worldwide (Weiser et al., 2015), with rates increasing globally (Vogel et al., 2015). Additionally, caesarean section is associated with the risk of moderate to significant bleeding. Obstetric haemorrhage remains an important cause of maternal mortality worldwide, accounting for over 44,000 premature deaths each year, especially in low- and middle-income countries (Kassebaum et al., 2014).

Anaemia is a common and, in some instances, modifiable perioperative risk factor in women undergoing caesarean section. A Hb concentration of less than or equal to 100 g/L, and a haematocrit of less than 32% are risk factors for maternal transfusion (Rukuni et al., 2016; Ahmadzia et al., 2018) and neonatal morbidity including preterm birth, low birthweight, and potentially perinatal mortality (Nair et al., 2016; Rahman et al., 2016; ACOG practice bulletin no. 95: anemia in pregnancy, 2008). Post-partum anaemia is also associated with fatigue, depression, and impaired mother-infant bonding (Milman, 2011). Anaemia, when it is due to iron deficiency, may be able to be diagnosed and treated prior to surgery, thereby mitigating some of these risks and reducing morbidity and possibly mortality in pregnant women and their babies.

The traditional definition of anaemia by the World Health Organization (WHO) of ((Hb < 110 g/L in pregnant women has been recently challenged (Butcher et al., 2017). A consensus statement published by Munoz and colleagues, who represent a group international experts, recommend that patients undergoing major surgery—including the obstetric population—have a target Hb level > 130 g/L, regardless of sex (Munoz et al., 2017). This recommendation means that, contrary to WHO definitions, pregnant women with Hb values between 110 and 129 g/L are now considered mildly anaemic. Global data from WHO in 2019 suggest that the prevalence of anaemia (Hb < 100 g/L) in all women undergoing childbirth is approximately 29.9%. However, the specific group of women undergoing surgery during pregnancy (i.e. caesarean section surgery) has been poorly examined (World Health Organization, 2022a). We aimed to determine the prevalence of perioperative anaemia—specifically iron deficiency anaemia—in women undergoing caesarean section using the Munoz and colleagues (Hb < 130 g/L) threshold for diagnosing anaemia.

## Methods

After institutional approval (The Royal Women’s Hospital HREC EC00259 AQA 17/29), we conducted a retrospective cohort study of 489 consecutive women who underwent caesarean section over a 12-week period in a single-centre tertiary referral maternity unit in Australia. Inclusion criteria comprised all women undergoing caesarean section, planned (elective) or unplanned (urgent and emergent), including women with a history of antepartum bleeding or other pre-existing medical conditions. Patients who underwent successful operative vaginal delivery were excluded. We calculated the proportion of women who met the contemporary definition of perioperative anaemia (Hb < 130 g/L) at four time points—first hospital appointment, third trimester, preoperatively, and on discharge from hospital. Serial haemoglobin concentration measurements were recorded at the first hospital appointment, third trimester, preoperatively, and postoperatively. We calculated the proportion of women with no anaemia (Hb ≥ 130 g/L) (group 1), mild anaemia (Hb 110–129 g/L) (group 2), and moderate anaemia (Hb 80–109 g/L) (group 3). Iron deficiency anaemia was defined as a haemoglobin concentration less than 130 g/L with a ferritin level of less than 30 μg/L (The Royal College of Pathologists of Australia, 2022). Iron deficiency was defined as ferritin values of less than 30 μg/L. From ferritin levels measured at first hospital appointment, we classified women with anaemia into iron deficiency and non-iron deficiency anaemia. We determined the proportion of women investigated and treated for iron deficiency perioperatively, and the method of treatment. Perioperative data collected included operative blood loss and transfusion rates. Primary postpartum haemorrhage was defined as a blood loss of 500 mL or more during caesarean section, in accordance with the Royal College of Obstetricians and Gynaecologists of the UK (Mavrides et al., 2016).

Data were analysed using SPSS Statistics Version 27 (IBM© SPSS© Statistics IBM Corporation 2020, Chicago, IL). Data are presented as mean, standard deviation (SD), median (quartiles) [lowest and highest value]), or number and percentage as appropriate. Comparisons between and within groups were performed using paired or two sample *t* tests, one-way ANOVA with Tukey’s multiple comparison test, Kruskal-Wallis test with Dunn’s multiple comparisons test, Fisher’s exact test, or chi-squared test, for proportions as appropriate. Mean differences, 95% confidence intervals (CI), and two-sided *P* values are given with a significance level alpha of 0.05.

Strength of evidence statements were used to correspond to *P* values: < 0.001 very strong evidence, 0.001 to < 0.01 strong evidence, 0.01 to 0.05 evidence, > 0.05 no evidence. Where relevant, the clinical significance of differences is stated.

## Results

**Fig. 1:**
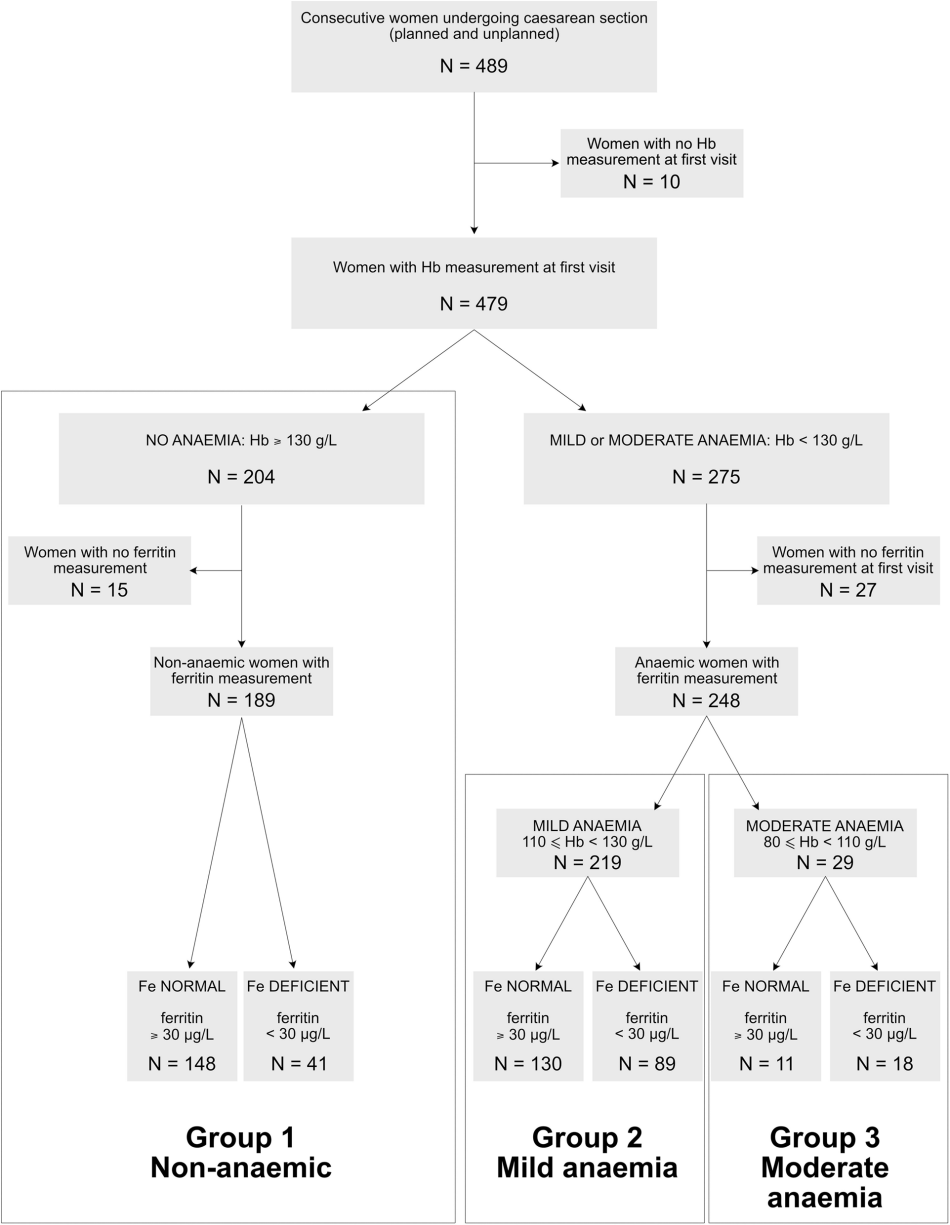
Participant flow chart and classification of anaemia in women undergoing caesarean section. This figure classifies women into one of three groups based on their first hospital appointment haemoglobin concentration : group 1—non-anaemic, group 2 mild anaemia, and group 3 moderate anaemia. Groups 1, 2, and 3 are further divided into those women with normal iron levels and those with iron deficiency. Sixteen of the women with no iron deficiency or anaemia had various pre-existing conditions and pregnancy related conditions (four women thalassemia, one woman idiopathic thrombocytopenic purpura, one woman a recent history of acute myeloid leukaemia, six women with twins, and four women with placenta praevia). Hb = haemoglobin; Fe = iron

Four hundred eighty-nine consecutive women underwent caesarean section during the 12-week period. Figure 1 shows the participant flow chart and distribution of anaemia and iron deficiency in the group at their first hospital appointment. Ferritin levels were measured in 437 women at their first hospital appointment. One hundred forty-eight (33.9%) women were iron deficient – 107 (72.3%) with anaemia, and 41 (27.7%) without anaemia. Of the 248 women with anaemia (Hb < 130 g/L) who had ferritin levels measured, iron deficiency was present in 107 (43.1%) women. This comprised 89 (40.6%) women with mild anaemia and 18 (62.1%) women with moderate anaemia. One hundred forty-one (56.9%) women were anaemic but not iron deficient. In this group, 130 (92.2%) women had mild anaemia and 11 (7.8%) women had moderate anaemia. One hundred forty-eight (33.9%) women had haemoglobin levels greater than or equal to 130 g/L with no iron deficiency. In the group of women with anaemia but no iron deficiency at first hospital appointment, 11 women subsequently were found to have low ferritin levels later in pregnancy. This observation suggests that their ferritin reserves were too low early in pregnancy to sustain adequate iron synthesis throughout pregnancy. The prevalence of anaemia from all causes using the WHO threshold of Hb 110 g/L and the threshold of 130 g/L was 6.9% and 57.4% at first hospital appointment, 26.1% and 94.0% in third trimester, 12.2% and 66.0% preoperatively. As only 202 women had postoperative Hb measured, the minimum hospital discharge anaemia prevalence was determined by assuming the missing 277 women all had Hb levels ≥ 130 g/L and dividing the number of women with recorded anaemia by the total sample size of 479. The minimum prevalence of postoperative anaemia was 40.3% using a threshold of 130 g/L and 23.3% using a threshold of 110 g/L.

**Fig. 2:**
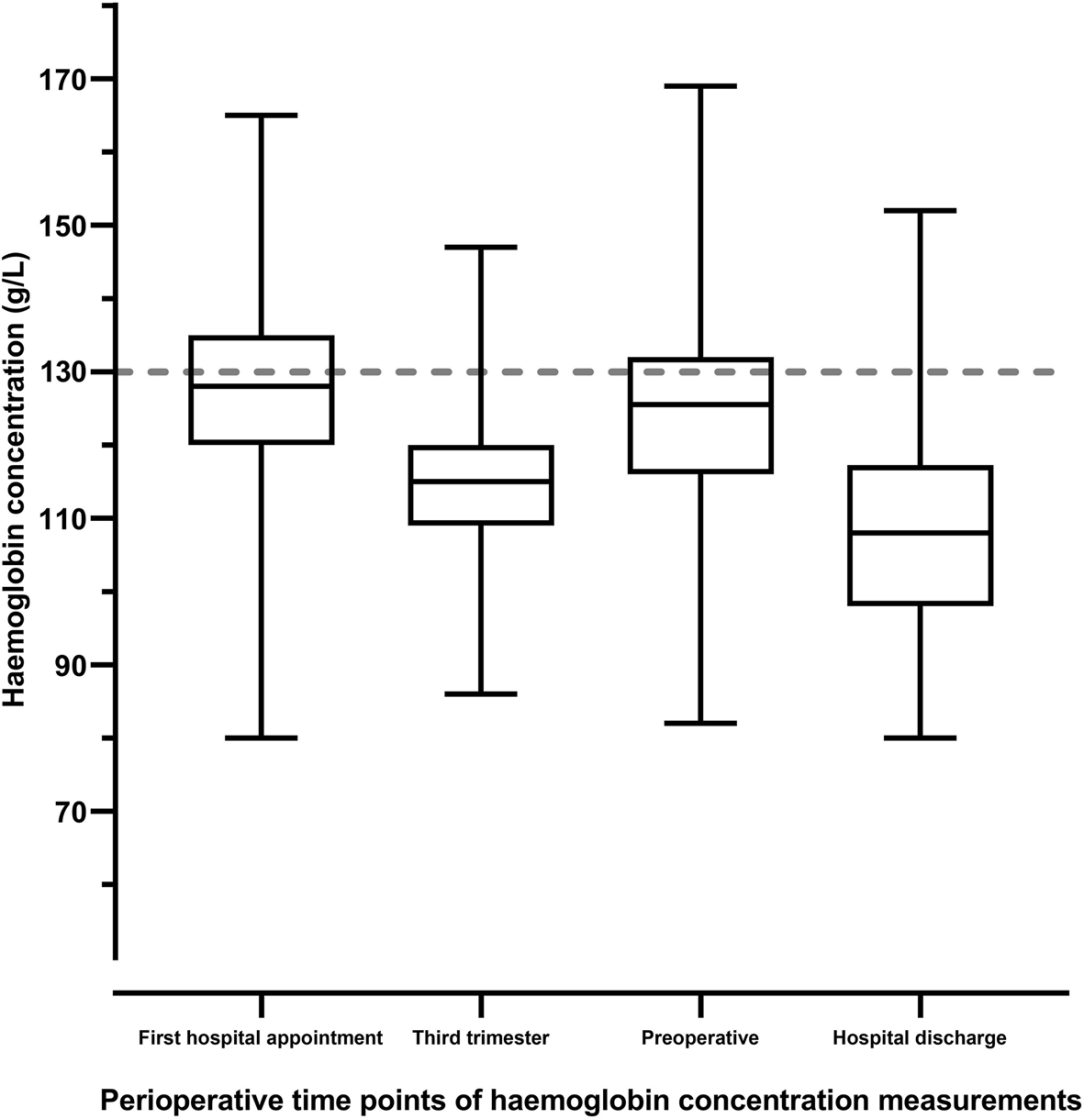
Box plots of haemoglobin concentration from the whole group taken at four timepoints. First hospital appointment timepoint corresponds to a gestation of 9.9 (7.28) weeks. The third trimester timepoint corresponds 27.6 (1.88) weeks. The preoperative timepoint corresponds to 37.9 (2.79) weeks. The horizontal line in each box represents the median value, the limits of the box represent the lower 25th centile and the upper 75th centile. The whiskers represent the lowest and highest values. The dashed line perpendicular to the *Y* axis is the line marking 130 g/L, the threshold value below which anaemia is defined. The small rise in haemoglobin and fall in prevalence of anaemia in the preoperative sample may be partly explained by the effects of fasting on haemoglobin concentration

Table 1 shows the participant characteristics of the whole group at their first hospital appointment as well as the whole group divided into three subgroups: group 1—women with no anaemia (haemoglobin concentration ≥ 130 g/L), group 2—women with mild anaemia (haemoglobin concentration 109–129 g/L), and group 3—women with moderate anaemia (haemoglobin concentration 80–109 g/L). No woman at any time point had a Hb < 80 g/L. 275 women, representing 57% of the total group, were anaemic at their first hospital appointment using a threshold of 130 g/L. By comparison, 33 women (6.9%) were moderately anaemic using a threshold of 110 g/L (Table 1, Fig. 2). Of the 275 women who were anaemic from all causes, only 90 (32.7%) received treatment for anaemia; 72 (29.8%) women with mild anaemia, and 18 (54.5%) women with moderate anaemia. Treatment included oral iron (61 women with mild anaemia, 15 women with moderate anaemia), and iron infusions (11 women with mild anaemia, three women with moderate anaemia). In the subgroup of 148 women who had iron deficiency (107 with anaemia, and 41 without anaemia, Fig. 1) at their first hospital appointment only 68 (45.9%) women received treatment. Of the 89 women with mild iron deficiency anaemia, 38 received oral iron and seven received iron transfusions. Of the 18 women with moderate anaemia, 12 received oral iron and two received iron infusions.

**Table 1:** Participant characteristics at first hospital appointment. Values are mean (SD), or number and percentage

Characteristic	Whole group	GROUP 1 No anaemia at first hospital appointment Hb ≥ 130 g/L N=204	GROUP 2 Mild anaemia at first hospital appointment Hb 110 - 129 g/L N=242	GROUP 3 Moderate anaemia at first hospital appointment Hb 80 - 109 g/L N=33	***P*** value*
First hospital appointment - Gestation (weeks)	9.9 (7.3)	6.8 (3.6)	11.4 (7.8)	18.4 (9.8)	<0.0001
First hospital appointment - Haemoglobin concentration (g/L)	126.7 (11.4)	137.0 (5.9)	121.2 (5.3)	103.2 (6.6)	N/A
First hospital appointment – Women with anaemia, Hb < 130 g/L (n,%)*	275/479 (57.4%)	0	242/479 (50.5%)	33/479 (6.9%)	N/A
Iron deficiency (ferritin <30 μg/L)	148/437(33.9%)	41/189 (21.7%)@	89/219 (40.6%)	18/29 (62.1%)	<0.0001
Twin pregnancy	24	9	12	3	N/A&
Abnormal placentation including placenta praevia	10	3	5	2	N/A&
Age (years)^#^	32.5 (5.2)	32.8 (5.1)	32.5 (5.2)	30.6 (5.4)	0.0780
First hospital appointment - Height (cm)	162.9 (7.44)	162.9 (7.7)	163.0 (7.5)	162.6 (5.1)	0.9625
First hospital appointment - Weight (kg)	70.1 (16.8)	70.6 (16.0)	70.0 (18.0)	68.6 (14.4)	0.8275
First hospital appointment - Body mass index (kg/m^2^)	26.3 (5.9)	26.5 (5.9)	26.2 (5.9)	26.0 (5.4)	0.8240

*Difference between Group 1,2 and 3

This table shows the data for the whole group, at first hospital appointment, with women divided into three groups based on first hospital appointment haemoglobin concentration measurement. In the anaemia groups, four women had thalassemias, and one woman had systemic lupus erythematosus.

*Four hundred eighty-nine women were included in the total group, however, 10 patients did not have a haemoglobin concentration measurement at first hospital appointment therefore anaemia proportion is out of 479 women. Ferritin levels were measured in 437 of these women.

N/A = not applicable as participants were divided into three groups depending on their first hospital haemoglobin concentration measurement so they are expected to be different.

#age at caesarean section.@ These 41 people were iron deficient without anaemia (ferritin < 30 μg/L). This is also shown in Fig. 1.

&numbers too small to compare

There was very strong evidence that women who were moderately anaemic had their first hospital appointment at more advanced gestations compared to women with no anaemia (mean difference 11.6 (0.9) weeks, 95% CI 9.7 to 13.4 weeks, *p* < 0.0001). The average body mass index at first hospital appointment was in the overweight range.

Table 2 shows the participant characteristics throughout pregnancy and postoperatively. By the third trimester, approximately 9 out of 10 women in the whole group (94%) were anaemic. The mean (SD) third trimester haemoglobin was 114.6 (10.6) g/L, and anaemia was present in 331 women (94.0%) using a threshold of 130 g/L, and 93 women (26.4%) using a threshold of 110 g/L.

**Table 2:** Participant characteristics in third trimester, pre-operatively, intraoperatively, postoperatively and at discharge from hospital. Values are mean (SD), or number and percentage

Characteristic	Whole group	GROUP 1 No anaemia at first hospital appointment Hb ≥ 130 g/L	GROUP 2 Mild anaemia at first hospital appointment Hb 1109 - 129 g/L	GROUP 3 Moderate anaemia at first hospital appointment Hb 80 - 109 g/L	***P*** value*
Third trimester - Gestation of haemoglobin measurement (weeks)	27.6 (1.87)	27.4 (1.7)	27.6 (1.9)	28.5 (2.4)	0.0212
Third trimester - Haemoglobin concentration (g/L)	114.6 (10.57)	119.2 (8.4)	112.2 (10.6)	104.5 (9.3)	<0.0001
Third trimester – Women with anaemia Hb <130 (n, %)	331/352^a^ (94.0%)	124/141 (87.9%)	179/183 (97.8%)	24/24 (100%)	0.0005
Preoperative - Gestation at caesarean section (weeks)	37.91 (2.79)	38.0 (2.8)	38.1 (2.6)	36.9 (3.4)	0.0835
Preoperative - Haemoglobin concentration (g/L)	124.10 (12.42)	128.2 (11.5)	122.4 (11.6)	111.5 (14.1)	<0.0001
Preoperative – Women with anaemia, Hb <130 (n, %)	318/482^b^ (66.0%)	108/202 (53.5%)	175/239 (73.2%)	27/31 (87.1%)	<0.0001
Preoperative - Weight (kg)	80.9 (16.80)	82.3 (15.9)	79.5 (17.7)	82.0 (16.0)	0.9959
Preoperative - Body mass index (kg/m^2^)	30.5 (5.83)	31.0 (5.7)	29.9 (5.9)	31.1 (5.7)	0.2958
Preoperative – Classified as emergency caesarean section (n, %)	271/486 (55.8%)	116/204 (57%)	131/239 (55%)	18/33 (54.5%)	0.9022
Intraoperative - Estimated blood loss (mL)	438.6 (341.7)	429.1 (225.9)	448.3 (428.8)	421.2 (236.2)	0.8049
Postoperative - Hospital length of stay (days)	4.1 (2.3)	4.1 (2.1)	4.1 (2.4)	4.3 (2.8)	0.901
Discharge - Haemoglobin concentration (g/L)	108.0 (13.6)	111.8 (13.0)	105.7 (13.4)	95.8 (11.9)	0.0001
Discharge - Women with discharge anaemia^c^, Hb <130 g/L (n, %)	193/202^c^ (95.5%)	82/88 (93%)	95/98 (98%)	10/10 (100%)	0.3036

*Difference between Group 1,2 and 3

This table shows serial data throughout the peri-operative period for the whole group as well as data for the three groups of women classified as having no anaemia, mild anaemia, or moderate anaemia at their first hospital appointment.

^a^352 women out of the whole group had third trimester haemoglobin concentration measured. Of the 10 women who did not have first antenatal appointment haemoglobin concentration measured, four had 3^rd^ trimester haemoglobin concentration measured, and all four women were anaemic (Hb < 130 g/L)

^b^Four hundred eighty-two women out of the whole group had pre-operative haemoglobin concentration measured. Of the 10 women who did not have first antenatal appointment haemoglobin concentration measured, all 10 had pre-operative haemoglobin concentration measured and eight women were anaemic (Hb < 130 g/L).

^c^Two hundred two women out of the whole group had discharge haemoglobin concentration measured. Of the 10 women who did not have first antenatal appointment haemoglobin concentration measured, six had discharge haemoglobin concentration measured and all six women had haemoglobin concentration <130 g/L.

There was very strong evidence of a difference in third trimester haemoglobin concentrations between group 1 (no anaemia at first hospital appointment), group 2 (mild anaemia at first hospital appointment) and group 3 (moderate anaemia at first hospital appointment). There was also very strong evidence that women who had mild or moderate anaemia at their first hospital appointment, compared to women who had no anaemia at their first hospital appointment, had lower third trimester haemoglobin concentrations (mean difference 7.1 (1.1) g/L, 95% CI of − 9.20 to − 4.91 g/L *P* < 0.0001, and mean difference 14.8 (1.9) g/L, 95% CI of − 18.50 to − 11.06 g/L P<0.0001 respectively).

Ferritin was measured at the first hospital appointment in 437 women (91.2%) at a mean (SD) gestation of 10.6 (8.1) weeks. Despite the mean (SD) preoperative ferritin of 57.7 (96.4) μg/L, 148 women (33.7%) had a preoperative ferritin < 30 μg/L. During pregnancy, only 84 women (17.1%) were treated with oral iron, and 18 women (3.7%) received an iron infusion (all during the third trimester).

Preoperative haemoglobin concentration was measured in 482 women at a mean (SD) gestation of 37.9 (2.8) weeks (Fig. 2). The mean (SD) preoperative haemoglobin was 124.1 (12.4) g/L. Preoperatively, two out of three women were anaemic (*n* = 318, 66%) having a haemoglobin concentration < 130 g/L, 163 women (33.8%) had a haemoglobin concentration < 120 g/L, and 59 women (12.2%) had a haemoglobin concentration < 110 g/L. The mean (SD) gestation at caesarean section was 37.9 (2.8) weeks, and over half of the women (*n* = 271, 55.8%) underwent emergency caesarean section. The mean (SD) intraoperative estimated blood loss was 438.6 (341.7) mL, and the mean (SD) decrease in haemoglobin concentration from before to after surgery was 16.1 (11.64) g/L. Five women (1.02%) required perioperative red blood cell transfusion.

There was very strong evidence that women who had mild and moderate anaemia at their first hospital appointment, compared to women who had no anaemia at first hospital appointment, had lower preoperative haemoglobin concentration levels (mean difference 5.8 (1.1) g/L, 95% CI − 7.79 to − 3.62 g/L *p* > 0.0001, and mean difference 16.6 (2.3) g/L 95% CI − 21.13 to − 12.14 g/L respectively).

Using a definition of postpartum haemorrhage (PPH) of 500 mL or greater blood loss (Mavrides et al., 2016), 140 (28.6%) women experienced a PPH with a mean (SD) blood loss of 752 (512.3) mL. Five women in the group (1.0%) received a blood transfusion. One of these women did not have a first hospital appointment haemoglobin concentration measurement, one had mild anaemia at first hospital appointment and three had moderate anaemia at their first hospital appointment.

Hospital discharge haemoglobin concentration was measured in 202 women (Fig. 2). One hundred ninety-three were anaemic (96%) with a mean (SD) discharge haemoglobin concentration of 108.0 (13.6) g/L. This comprised 82 women with no anaemia at their first hospital appointment, 95 women with mild anaemia at their first hospital appointment, and 10 women with moderate anaemia at their first hospital appointment (Table 2). Of the 202 women who had a discharge haemoglobin measured, 193 (95.5%) had values < 130 g/L, and 112 women (55.4%) had values < 110 g/L, Of the 112 women with hospital discharge Hb < 110 g/L, 35 (31.3%) women were iron deficient at their first hospital appointment. This suggests that up to 30% of postoperative anaemia may be prevented with aggressive treatment of iron deficiency in early pregnancy.

There was strong evidence that women who had mild anaemia at their first hospital appointment, compared to women who had no anaemia at their first hospital appointment, had lower discharge haemoglobin concentration levels (mean difference 6.1 (1.9) g/L, 95% CI − 9.88 to − 2.23 g/L *P* = 0.0021). There was also very strong evidence that women who had moderate anaemia at their first hospital appointment, compared to women who had no anaemia at their first hospital appointment, had lower discharge haemoglobin concentration levels (mean difference 16.0 g/L, 95% CI − 24.55 to − 7.44 g/L *P* = 0.0003).

Only 53 women (27.5%) received treatment on discharge for their anaemia. Most women received oral iron supplements, with a small number of women receiving parenteral iron therapy (iron carboxymaltose infusion). The mean haemoglobin concentration of women receiving treatment on discharge was 97.9 (11.0) g/L.

## Discussion

A contemporary proposed definition of anaemia advocated by Munoz and colleagues is Hb < 130 g/L (Munoz et al., 2017). Using this definition, we demonstrated a high prevalence of iron deficiency anaemia perioperatively in women undergoing caesarean section in a high-income country. Over one in three women were iron deficient at their first hospital appointment, and nearly one in two women with anaemia had iron deficiency. Despite these large numbers, only 68 (46%) women received treatment; 57 (84%) receiving oral iron, and 11 (16%) receiving iron infusions.

Using a Hb threshold of 130 g/L, our findings demonstrate that at all timepoints in pregnancy the prevalence of anaemia was at least 50%. In the postpartum period on discharge from hospital, the prevalence was over 40%. Importantly, nearly one in three women who were anaemic by WHO standards on discharge from hospital were iron deficient at their first hospital appointment. This finding suggests that more aggressive treatment of iron deficiency in the antenatal period is needed to reduce significant postoperative anaemia after caesarean section.

Haemoglobin values decreased and anaemia prevalence increased with gestation. A small rise in haemoglobin concentration and a small fall in anaemia prevalence was noted preoperatively. This may be explained by the known reduction in dilutional anaemia at term (Milman et al., 2007), and possibly by preoperative fasting. Preoperatively, two out of every three women were anaemic. Postoperatively, almost all women were anaemic using a threshold of < 130 g/L, and over half of the women had a discharge haemoglobin less than 110 g/L. Similarly, only a few women with postoperative anaemia received treatment with intravenous iron. Our data also demonstrated a 6.4% rate of moderate postoperative anaemia (Hb < 90 g/L) and this was consistent with previously published data reporting severe anaemia in 7% of women after caesarean section (Butwick et al., 2017). We have also shown that postoperative anaemia after caesarean section is a significant issue. WHO classifies anaemia prevalence cut-off values for public health significance as < 5% no public health problem; 5–19% mild public health problem; 20–39% moderate public health problem; ≥ 40% severe public health problem. In this study we have shown the prevalence of postoperative anaemia in women having had a caesarean section, to be at least 25% of the total cohort. This represents a moderate public health problem. As women who are discharged from the hospital after caesarean section are no longer pregnant, non-pregnant Hb reference ranges should apply to them. With such a high prevalence of postoperative anaemia, future studies should serially measure Hb levels in the weeks to months after caesarean section to determine changes in haemoglobin in this population (World Health Organization, 2022b).

Caesarean section is a moderate-to-high blood-loss open abdominal surgery, with an average blood loss of 440 mL to 800 mL (Stafford et al., 2008; Larsson et al., 2006), and associated transfusion rates of 2 to 4% (Hammad et al., 2014; Ferguson & Dennis, 2019). A physiologic dilutional anaemia of pregnancy occurs during the second trimester and the haemoglobin drop associated with elevated plasma volume has recently been demonstrated to be greater than previously reported (14 g/L compared to 5 g/L) (Churchill et al., 2019; World Health Organisation, 2011). This may contribute to perioperative anaemia if pre-pregnancy haemoglobin concentrations are in the anaemic range.

Antenatal anaemia is common. According to WHO, anaemia is present in 38% of pregnant women globally (Stevens et al., 2013). 39% of all women undergoing caesarean section in a large healthcare network in the USA (Butwick et al., 2017) were found to be anaemic. Among women receiving routine iron supplementation during pregnancy, the mean haemoglobin concentration at term ranges from 124 g/L to 129 g/L (Milman et al., 2007; Centers for Disease Control and Prevention (CDC), 1989; Milman et al., 2000).

The antenatal period therefore represents an important window of opportunity for haemoglobin optimisation (Wilson et al., 2018; Munoz et al., 2018a). Australian and UK guidelines recommend measuring haemoglobin concentration at the first hospital appointment and at 28 weeks gestation (Royal Australian and New Zealand College of Obstetricians and Gynaecologists, 2022; Royal College of Obstetricians and Gynaecologists, 2015). These time-points provide convenient windows for an initial trial of oral iron during the first trimester, and intravenous iron if anaemia persists at 28 weeks. Oral iron supplementation reduces the incidence of maternal anaemia at term by approximately 50 to 70% (Haider et al., 2013; Pena-Rosas et al., 2015), and increases haemoglobin concentration values by 8.9 g/L at term and 7.6 g/L at 6 weeks postpartum (Pena-Rosas et al., 2015). There are also trends towards a reduced incidence of low birthweight and preterm birth (Haider et al., 2013; Pena-Rosas et al., 2015). Antenatal haemoglobin optimisation may also reduce transfusion rates after caesarean and vaginal delivery (Flores et al., 2017).

Serum ferritin levels below approximately 100 μg/L represent iron stores that are inadequate for postoperative haematopoiesis (Munoz et al., 2018b). Additionally, 75 to 80% of pregnant women have absent postpartum bone marrow iron stores without iron supplementation (Puolakka et al., 1980; De Leeuw et al., 1966) due to the high net additional iron requirement of pregnancy and delivery (approximately 1000 mg) (Puolakka et al., 1980). Postoperatively, hepcidin is upregulated; consequently, oral iron is poorly absorbed (Munoz et al., 2018b). Therefore, postpartum anaemia after caesarean section will likely persist in the absence of postoperative treatment with intravenous iron. Despite this, only 13.1% of patients in our study received oral iron postoperatively, and only 1.2% received a postoperative iron infusion. Postpartum anaemia has important effects on maternal quality of life including being associated with a higher burden of depressive symptoms (Corwin et al., 2003) and adverse mother-infant interactions (Perez et al., 2005).

Specifically related to anaemia, perioperative anaemia in women undergoing caesarean section represents an important global health issue, particularly in low-income countries where iron deficiency anaemia is prevalent and caesarean section accounts for almost one-third of all operations performed.

Beyond the emphasis on caesarean section as an alternative mode of birth, there is an urgent need to raise the profile of caesarean section as a moderate risk surgery associated with the significant risk of blood loss (Dennis & Sheridan, 2019). There is a critical need to identify, classify, and treat anaemia in the early antenatal period (first hospital appointment) to decrease the prevalence of preoperative, and postoperative anaemia. The key to increasing awareness is the acceptance and acknowledgement of all individuals involved in the care of pregnant women. Also, women must be made aware that iron deficiency anaemia is highly prevalent after caesarean section and can occur in one in three pregnant women. Society can then begin to have informed discussions that consider the antenatal period as an essential time for not only preparing for the birth and care of a baby, but a time for the preparation of women for major surgery. Furthermore, essential data must be collected that includes the measurement of haemoglobin concentration and ferritin in the perioperative period including at the first hospital appointment, during third trimester, preoperatively and postoperatively. Additional iron studies including transferrin saturation, vitamin B12 and folate should be collected to determine the cause of non-iron deficiency anaemia. Despite numerous studies surrounding anaemia, there is still significant uncertainty as to what the appropriate reference range for haemoglobin should be in healthy pregnant women. This uncertainty arises because pregnancy is a unique situation compared to all other abdominal surgeries, in that the body is physiologically prepared to accommodate some level of blood loss in the healthy pregnant woman. Munoz and colleagues have recommended a preoperative Hb of 130 g/L or more in all patients. However, additional well-designed studies are needed to determine whether this is an appropriate level in the healthy pregnant woman with or without iron deficiency. Serial studies well into the postpartum period are also needed to observe the changes in haemoglobin concentration after recovery from pregnancy.

There is also a need to develop and use more precise measurements of intraoperative blood loss, such as quantitative assessment measures which use gravimetric (weight-based), volumetric (volume-based), and colorimetric (image analysis of surgical sponges and cannisters) approaches.

Our study has several limitations. These include the retrospective nature of data collection and missing data, the lack of follow up beyond discharge from hospital (which may have shown a stabilisation and perhaps increase in haemoglobin concentration), and some imprecision in the reported data (such as estimated blood loss). In addition, the use of a retrospective study design increases the possibility of unmeasured confounders, and the use of a single institution limits the generalisability of our findings to the general patient population. Not all women had a haemoglobin concentration measured at each time point, and this limits the conclusions that can be drawn regarding postoperative anaemia prevalence, as only 202 women (41%) had a discharge haemoglobin measurement. However, even if the remaining non-sampled women all had normal haemoglobin concentration values, this still leaves almost half of all women anaemic on discharge postoperatively.

Importantly, we do not present outcome data. There is, however, some evidence in the literature that anaemia in pregnancy is related to poorer outcomes in both the woman and neonate. Future studies should explore the relationship between anaemia and patient centred outcome measures in this population. Outcomes such as blood transfusion rate, postoperative infection, postoperative hypertension, depression, fatigue, bonding with baby, and breastfeeding, would be important to correlate with haemoglobin concentrations.

## Conclusion

Our data strongly suggests that iron deficiency remains a leading but modifiable cause of moderate anaemia in pregnant women preoperatively, and postoperatively at hospital discharge. Our data suggests that intensive treatment of iron deficiency at the first hospital appointment may reduce the prevalence of postoperative anaemia by 30%. Further education should focus on the recognition and treatment of iron deficiency anaemia in pregnant women. Future research should focus on robust multicentre prospective studies that measure key maternal and neonatal outcomes stratified for haemoglobin concentrations and ferritin levels. These studies should include healthy women who are not iron deficient at their first hospital appointment in order to define physiological haemoglobin concentration, and reference ranges, during and after pregnancy, matched with pertinent clinical outcomes. Evidence-based definitions of perioperative haemoglobin concentration and optimisation in pregnant women will pave the way to further address other causes of anaemia in this patient population and reduce the incidence of maternal or neonatal morbidity and mortality attributed to this cause.

## Data Availability

On request.
